# House Dust Mite Specific Antibodies induce Neutrophilic Inflammation in the Heart

**DOI:** 10.7150/thno.47134

**Published:** 2020-07-09

**Authors:** Xiao Chen, Yuan-Yi Zhang, Dongting Ye, Gui Yang, Yan-Nan Song, Li-Hua Mo, Ping-Chang Yang, Jiang-Ping Song

**Affiliations:** 1 State Key Laboratory of Cardiovascular Diseases, Beijing Fuwai Hospital, Beijing, China.; 2 Research Center of Allergy & Immunology, Shenzhen University School of Medicine, Shenzhen, China.; 3 Guangdong Provincial Key Laboratory of Regional Immunity and Diseases, Shenzhen, China.; 4 Guangzhou First People's Hospital, School of Medicine, South China University of Technology.; 5 Department of Otolaryngology, Longgang Central Hospital. Shenzhen, China.; 6 Department of Pediatric Otolaryngology, Shenzhen Hospital, Southern Medical University, Shenzhen, China.

**Keywords:** heart, inflammation, neutrophil, autoimmunity, HDM

## Abstract

**Rationale**: Inflammatory heart disorders are among the causes of human death. The causative factors of heart inflammation are to be further elucidated. House dust mite (HDM)-derived protein antigens are involved in the pathogenesis of many human diseases. This study aims to investigate the role of HDM-specific autoantibodies in the pathogenesis of heart inflammation.

**Methods**: Human heart tissue samples were obtained from surgically removed hearts in heart transplantation. The interaction of the heart tissues with HDM-specific antibodies was assessed by pertinent immune analysis. The role of HDM-specific autoantibodies in the induction of heart inflammation was assessed with a murine model.

**Results**: HDM-specific IgG (mIgG) was detected in the serum of patients with myocarditis (Mcd); the mIgG titers were positively correlated with the neutrophil counts in the heart tissues. The mIgG specifically bound to keratin-10 (KRT10) in heart vascular endothelial cells and the heart tissue protein extracts. The amounts of C3a, C5a and C5b-9 were increased in the mouse heart tissues after exposing to mIgG. In the presence of the complement-containing serum, mIgG bound cardiovascular epithelial monolayers to impair the barrier functions. Administration of mIgG or HDM induced the Mcd-like inflammation in the heart, in which neutrophils were the dominant cellular components in the infiltration of inflammatory cells.

**Conclusions**: Mcd patients with neutrophilic inflammation in the heart had higher serum levels of mIgG. The mIgG bound heart endothelial cells to impair the endothelial barrier functions and induce neutrophilic inflammation in the heart.

## Introduction

Although heart inflammation is not a common disease in the clinic, it is frequently encountered in autopsy. Autopsy reports have revealed varying incidence of myocarditis, with estimates ranging from 0.12% to 12% in patients died of other diseases 1, 1, 2, suggesting that the inflammatory heart disorders may not be a rare condition. Myocarditis (Mcd) is one type of inflammatory heart diseases; its pathogenesis is not fully understood yet 3. If clinical intervention is not properly carried out for Mcd, serious complications may occur, such as the heart ventricular dilation or heart failure 4, 5. The therapeutic effects of Mcd is currently unsatisfactory 6. Therefore, it is necessary to further investigate the Mcd pathogenesis.

It is the consensus that Mcd is an immune disease 7, 8. Pathological assessment shows profound infiltration of immune cells, including lymphocytes, macrophages, eosinophils and neutrophils, in the inflamed area of the Mcd heart tissues 9 5 4. Although the role of each immune cell type in the inflammation is relatively clear, factors gathering immune cells to the inflammatory spot in the heart tissues remain elusive. Recurrent attacks are another Mcd clinical feature, the underlying mechanism is incompletely understood yet.

It is known that the streptococcal infection may complicate rheumatic heart diseases 10. This is because the immune system recognizes the streptococcal antigens, produced specific antibodies. These antibodies can attack the streptococcal bacteria as well as the heart tissues because some protein sequences of the heart tissues are similar to the bacteria; the antibodies are designated autoantibodies 10. House dust mites (HDM) are also associated with the pathogenesis of many human diseases, e.g., allergic asthma, allergic rhinitis, and allergic dermatitis 11, 12. By inducing the specific IgE antibody, HDM-derived antigens finally activate mast cells to induce aberrant immune responses and inflammation in the local tissues 11. HDM antigen-induced IgG may have protective functions 13 or induce abnormal immune responses 14, 15. The HDM-specific IgE and IgG can be detected in the serum and used as canonical parameters for diagnosing immune inflammatory diseases, such as asthma 16. This implies that, once produced in the body, HDM-specific antibodies can naturally reach the cardiovascular system including the heart. It is known that the antibody/antigen reactions are among the inflammatory causative factors 17, 18. 18, 19. Yet, whether the HDM-specific antibodies are involved in the Mcd pathogenesis has not been investigated.

Besides inducing allergic diseases, HDM antigens are also involved in the pathogenesis of other inflammatory disorders, e.g., intestinal inflammatory lesion 20, arthritis 21 and diabetes 22. This inspired us to search the probability that HDM-derived antigens might be associated with heart immune diseases since HDMs distribute in human living environment extensively and easily get into the human body 11, 12. Therefore, in the present study, we assessed the HDM-specific IgG antibodies (mIgG) in Mcd patients. The influence of mIgG on cardiovascular endothelial barrier functions and induction of inflammation in the heart was investigated with a murine model.

## Materials and Methods

### Collection of human explant hearts

Human explant hearts were obtained from the operation facility of Beijing Fuwai hospital after the heart transplantation surgery. The demographic data of the patients are presented in Table 1. The inflammatory types of the hearts were evaluated by pathologists. Patients with any of the following conditions were excluded from the present study: Cancer, allergic diseases, autoimmune diseases of other organs or under treatment with immune suppressors for any reasons, based on the disease history, routine clinical examinations and clinical laboratory tests. A written informed consent was obtained from each human subject. The experimental procedures were approved by the Human Ethical Committee at Beijing Fuwai Hospital. The investigation conforms to the principles outlined in the Declaration of Helsinki”.

### Induction of inflammation in the heart by mIgG

C57BL/6 mice were treated with mIgG (20 µg/mouse in 0.1 ml saline; control mice received isotype IgG) through tail vein injection daily for 6 days. Mice were sacrificed on day 7. Heart endothelial barrier functions were tested; the ECG was recorded before the sacrifice. The hearts were excised to be examined for the following parameters: Histology, immunohistochemistry to illustrate apoptotic endothelial cells and complements surrounding the endothelium, frequency of inflammatory cells and levels of inflammatory cytokines in the heart tissues.

### Induction of inflammation in the heart by HDM

C57BL/6 mice were immunized by subcutaneously injecting with HDM (Wowu Biotech, Huangzhou, China) at 0.1 mg/mouse mixed in 0.1 ml complete Freund adjuvant on the back skin. The injection was repeated on day 7 and day 14, respectively. From day 21, mice were challenged by intraperitoneally injection with HDM at 0.1 mg/mouse mixed in 0.1 ml incomplete Freund adjuvant every 3-day for 10 times. Mice were sacrificed 3 days after the last injection. The heart was removed and processed to assess inflammation in the heart as described above. ECG was recorded one day before the sacrifice. Control mice were injected with saline.

### Statistics

Data are presented as mean ± SEM. The difference between two groups was determined by the Student *t-*test. ANOVA followed by the Tukey's multiple comparison test or Bonferroni test was performed for multiple comparisons. P<0.05 was set as a significant criterion.

Some experimental procedures are presented in supplementary materials.

## Results

### Neutrophil counts in the Mcd heart tissues are positively correlated with serum titers of HDM-specific IgG

**Figure 1:**
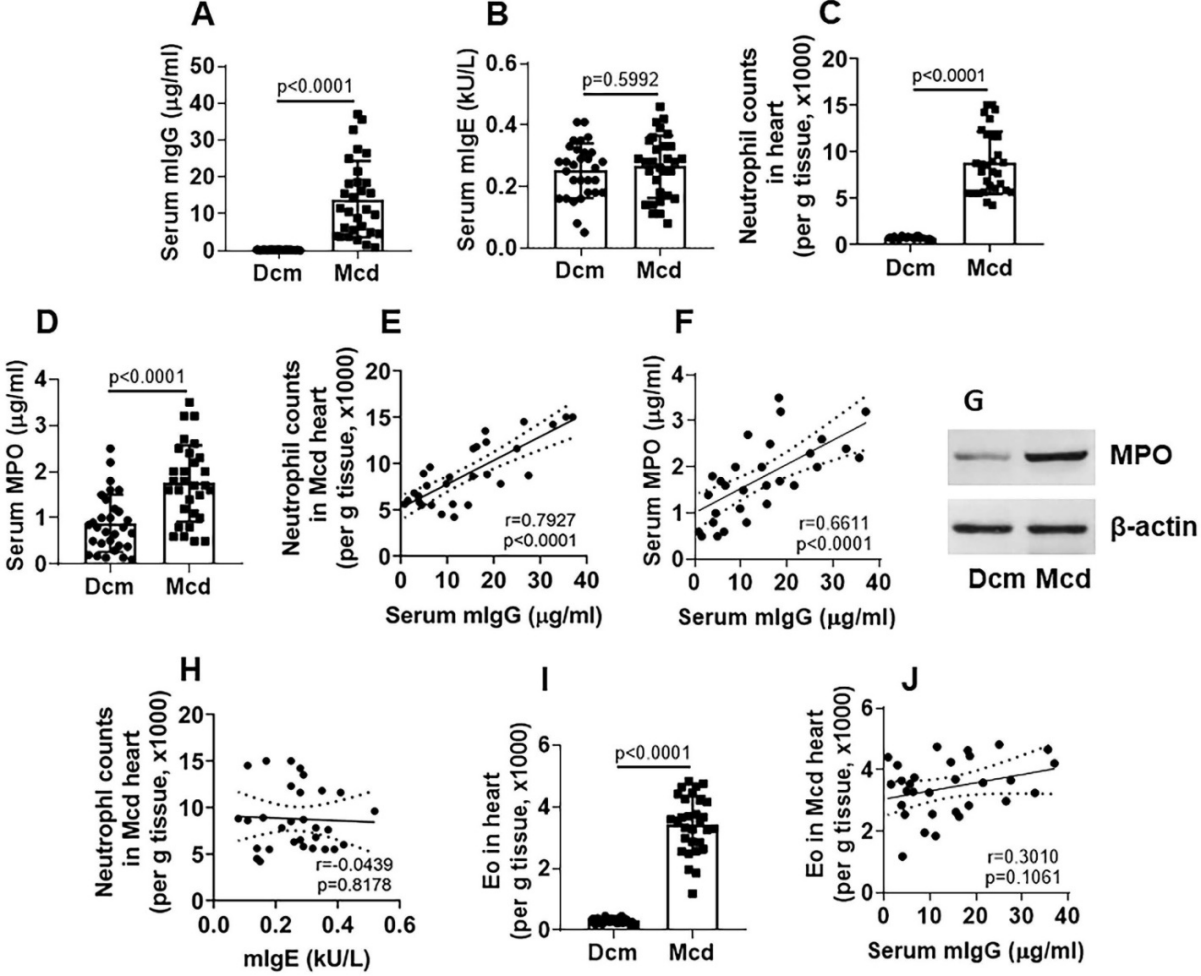
** neutrophil frequency is positively correlated with serum mIgG levels in advanced Mcd hearts**. Explant heart tissues were collected from myocarditis (Mcd) patients (n=30) and dilated cardiomyopathy (Dcm) patients (n=30). Serum mite-specific IgG (mIgG) and mIgE levels were determined. Mononuclear cells were isolated from the heart tissues and analyzed by flow cytometry to assess the frequency of neutrophils and Eos. **A-B**, serum mIgG (A) and mIgE (B) levels. **C**, counts of neutrophil in the heart tissues. **D**, serum MPO levels. **E-F**, positive correlation between serum mIgG and Mcd neutrophil (E) or serum MPO (F). **G**, immunoblots show MPO protein levels in heart tissue protein extracts. **H**, no correlation between neutrophil and mIgE. **I**, Eo counts in the heart tissues. **J**, no correlation between serum mIgG and Mcd Eo counts. The data of bars are presented as median (IQR). Each dot in bars presents data obtained from one sample. Statistics: The Mann Whitney test (A, B, C, D, G) and Pearson correlation assay (E, F, H, J).

To search the probability that HDM-derived factors might linked to the Mcd pathogenesis, we investigated the association between the inflammatory cells (neutrophil and Eo) in the heart tissues and HDM-specific antibodies (mIgG and mIgE) in patients with end stage of Mcd, who had undergone the heart transplantation. We found that, compared to patients with non-Mcd dilated cardiomyopathy (Dcm), the serum titers of mIgG (Figure 1A), but not mIgE (Figure 1B), were significantly higher in Mcd patients. Mononuclear cells were isolated from the heart tissues; inflammatory cells, including neutrophils and eosinophils (Eo), were counted by flow cytometry. The results showed that the number of neutrophil (Figure 1C) and MPO (Myeloperoxidase; an indicator of neutrophil activities) (Figure 1D) were generally higher in the hearts of Mcd patients, which positively correlated with the serum mIgG titers (Figure 1E-F). High MPO levels were also found in heart tissue protein extracts (Figure 1G). No correlation was found between serum mIgE and heart neutrophil counts (Figure 1H). The counts of heart Eos were also higher in the Mcd group than those in the Dcm group (Figure 1I), but no correlation was detected between heart Eo counts and serum mIgG (Figure 1J). The results demonstrate that Mcd patients have high serum mIgG titers which are positively correlated with the neutrophil counts in the heart tissues. The results imply that the neutrophil infiltration in the heart tissues and the high serum mIgG titers may be associated with the Mcd pathogenesis.

### mIgG specifically recognizes the constitutive protein KRT10 in the heart tissues

**Figure 2:**
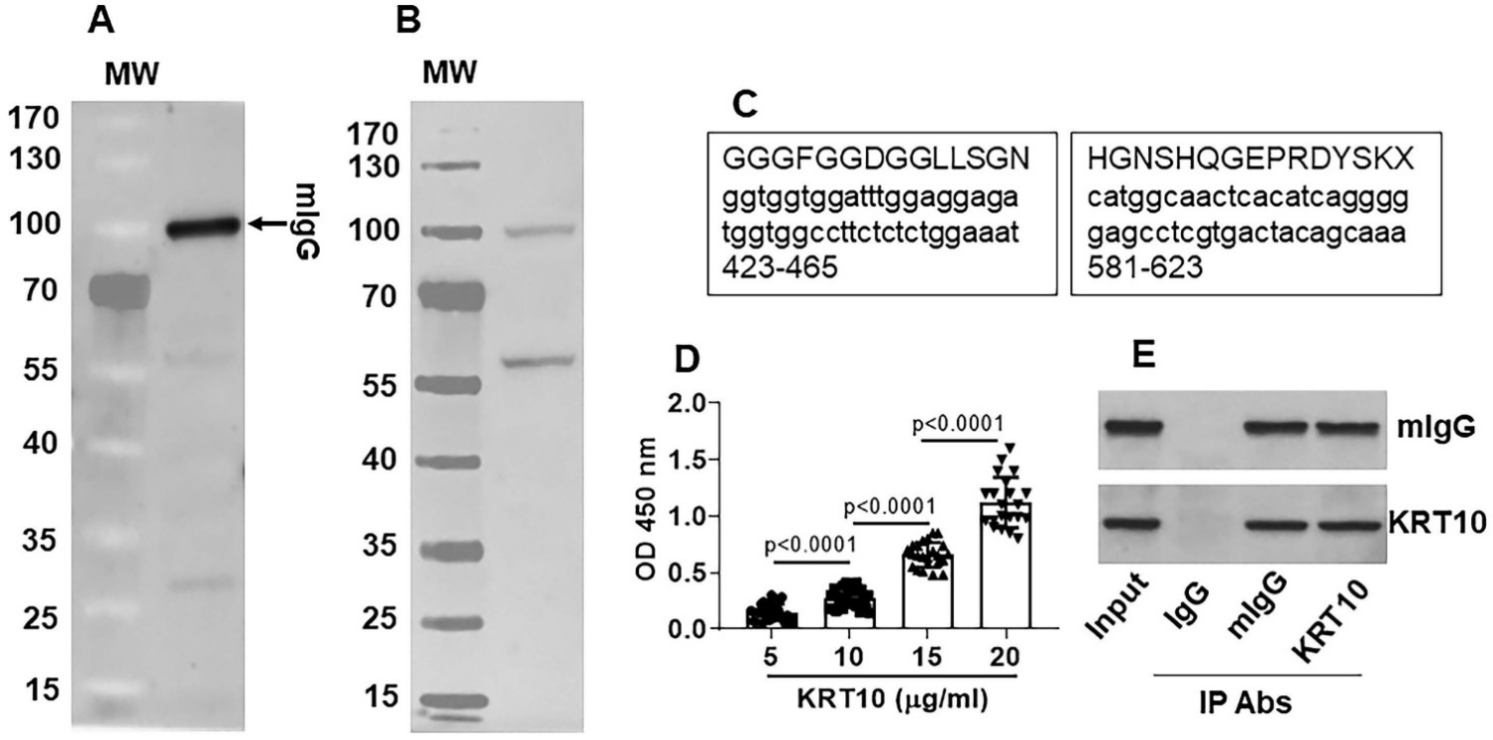
** mIgG recognizes the KRT10 protein in the Mcd heart. A**, mIgG was purified by affinity chromatography. The immunoblot shows purified mIgG. **B**, protein extracts were prepared with the explant heart tissues (n=30). The proteins were mixed with purified mIgG for 30 min. The protein bands in gel graph were the immune complexes that were precipitated by protein G and separated by SDS-PAGE. **C**, the protein about 55-60 kDa in panel B was analyzed by mass spectrometry (MS). The MS results show the protein is KRT10. The schemes show two representative KRT10's amino acid sequences (the upper cases) and the corresponding gene sequences (the lower cases; the numbers show the sequence loci in the gene. **D**, the serum of Mcd patients (n=30) were analyzed by ELISA with mIgG as the antibody and KRT10 (purified from the heart tissues) to coat the plates at gradient concentrations. The bars show KRT10-specific IgG in the serum. **E**, protein extracts were prepared with the Mcd heart tissues and analyzed by immunoprecipitation (IP) with mIgG and anti-KRT10 antibody as the IP antibodies. The immunoblots show immune complexes of mIgG and KRT10 in the Mcd heart tissues. The data of bars in panel D are presented as mean ± SEM. Statistics: ANOVA + Bonferroni test. The data of panels A, B, C and E are from one experiment that represent 3 independent experiments.

By affinity chromatograph approach, mIgG was purified from the serum obtained from Mcd patients (Figure 2A). The purified mIgG was mixed with proteins extracted from the explant heart of the same patients and incubated with mIgG to form complexes of mIgG/heart tissue proteins. The complexes were isolated by protein G precipitation and separated by SDS-PAGE. A protein about 55-60 kDa was identified in the complex with mIgG (Figure 2B). The protein was analyzed by mass spectrometry. The results showed that KRT10 was the protein bound by mIgG (Figure 2C). The results were verified by analyzing the proteins with ELISA (Figure 2D) and immunoprecipitation (Figure 2E). The results demonstrate that serum-isolated mIgG can recognize and form complexes with KRT10 protein in the heart.

### mIgG binds cardiovascular endothelial cells

**Figure 3:**
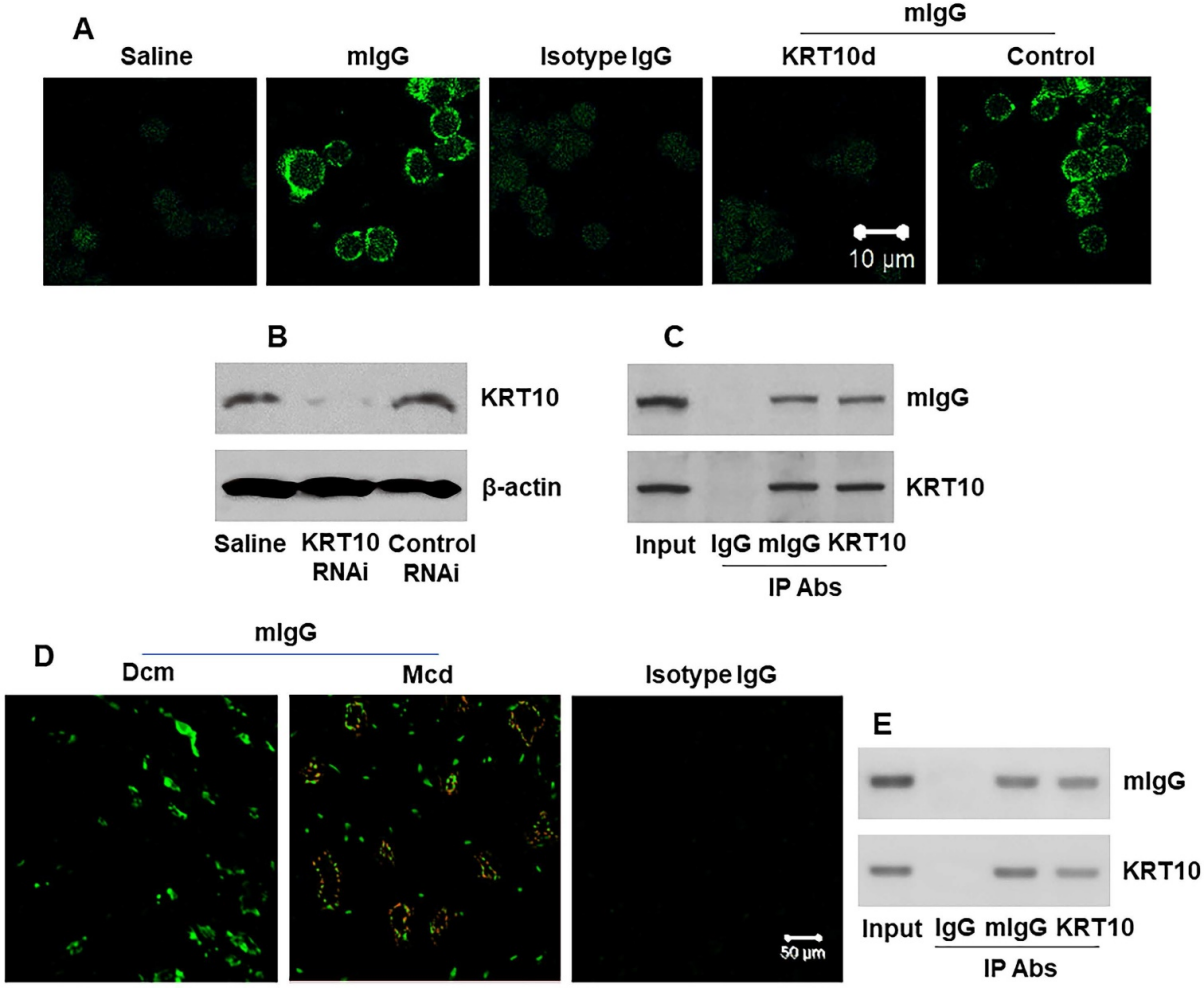
** mIgG binds KRT10 in vascular endothelial cells and heart tissues. A**, representative images (original magnification: ×630) show mIgG positive staining (in green) on the surface of HUVECs. KRT10d: KRT10-deficient HUVECs (made by RNAi). Control: HUVECs were treated with control RNAi reagents. **B**, KRT10 RNAi results. **C**, IP results show the mIgG/KRT10 complexes in HUVECs after exposing HUVECs to mIgG in the culture. **D**, representative images (original magnification: ×400) show mIgG (in red) and KRT10 (in green) staining in the human heart tissues. **E**, IP results show a complex of mIgG and KRT10 in protein extracts of the heart from mice treated with mIgG by tail vein injection. The data represent 6 independent experiments.

Next, we generated polyclonal mIgG (Figure S1 in supplemental materials). Human umbilical vein endothelial cells (HUVECs) were smeared on slides and stained with mIgG. We found that mIgG bound on the surface of HUVEC, but not the KRT10-deficient HUVECs (Figure 3A-B). The results were verified by IP, and a complex of mIgG/KRT10 was detected in HUVEC-extracts after exposing to mIgG in the culture (Figure 3C), but not in the saline controls (not shown). On the other hand, the human heart tissue sections were processed by confocal microscopy. We observed that the mIgG positive staining in the heart; and many endothelial cells were stained positive in Mcd sections, but not in Dcm sections (Figure 3D). In addition, mIgG was injected into naive mice via the tail vein daily for 7 days. The heart tissues were processed by IP. The results showed that mIgG bound KRT10 to form complexes (Figure 3E). Furthermore, we also found the CD31^+^ endothelial cells in the heart 23 expressed KRT10 (Figure S3). The results indicate that mIgG can specifically bind KRT10 in vascular endothelial cells in the heart.

### mIgG impairs endothelial barrier functions in the heart

**Figure 4:**
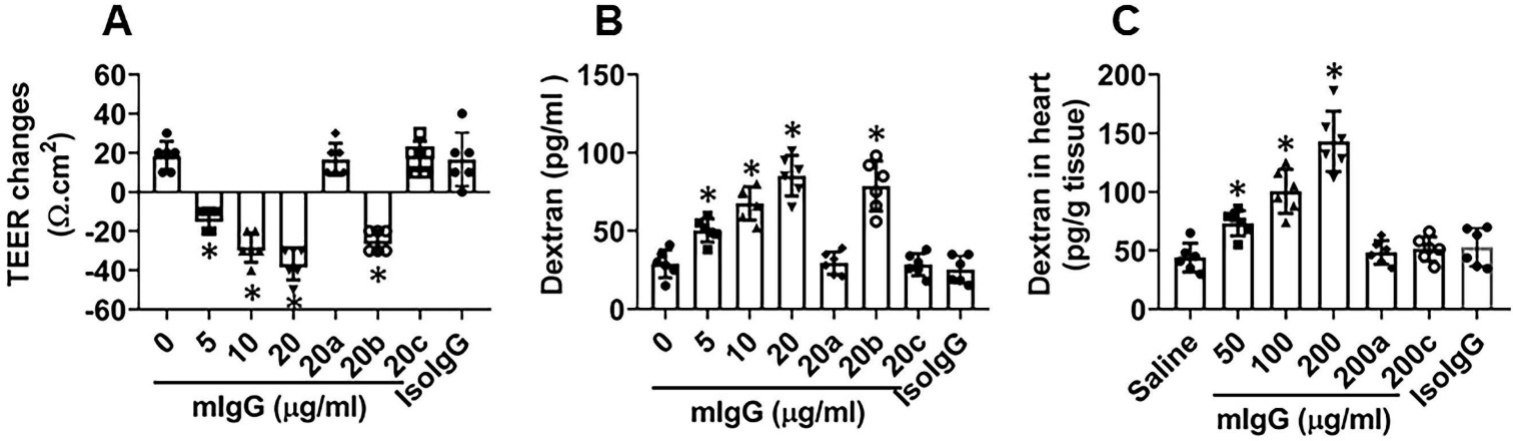
** mIgG impairs cardiovascular endothelial barrier integrity. A-B**, HUVEC monolayers were exposed to mIgG in Transwell system. A, TEER changes (against the TEER at the beginning time point) of HUVEC monolayers after exposing to mIgG in the culture for 16 h. B, dextran in the medium of the basal chambers of Transwells after exposing to mIgG or isotype IgG (isoIgG) in the culture for 16 h. **C**, dextran in the mouse heart tissues after treating with mIgG daily for 7 days. a, HUVEC monolayers deficient of KRT10. b, HUVEC monolayers were treated with control RNAi reagents. c, the serum was heated to quench complements. Data of bars are presented as mean ± SEM. Each dot in bars presents data obtained from an individual sample. Statistics: ANOVA + the Tukey's multiple comparison test. The data represent 6 independent experiments. In panel C, each group consists of 6 mice. *p<0.01, compared to the “0” group.

To test if mIgG affects endothelial barrier functions, a vascular endothelial monolayer model was developed. mIgG was added to the culture medium in the presence of the complement-containing serum. This induced a large drop of the transepithelial electric resistance (TEER) of the endothelial monolayers and significantly increased the permeability to macromolecular tracers, which could be blocked by knocking down the KRT10 expression (Figure 4A-B). To test the role of complements in the mIgG-induced endothelial barrier dysfunction, the serum was heated to quench the activities of complement before adding to the culture. Indeed, the effects of mIgG on impairing endothelial barrier function were abolished (Figure 4A-B). The results suggest that, in the presence of complements, mIgG can impair vascular endothelial barrier functions. Such an event was reproduced in an *in vivo* experiment. By treating mice with mIgG via tail vein injection daily for 7 days and using FITC-dextran as a tracer of the barrier permeability, the treatment with mIgG markedly increased the permeability of the vascular endothelial barrier in the heart, which was abrogated in KRT10-deficient mice or the presence of CVF (cobra venom factor; an inhibitor of complements 24) (Figure 4C); exposure to isotype IgG did not alter the endothelial barrier functions (Figure 4A-C). The results thus demonstrate that exposure to mIgG impairs the vascular endothelial barrier functions in the heart through interacting with KRT10.

### mIgG activates complements in the heart

**Figure 5:**
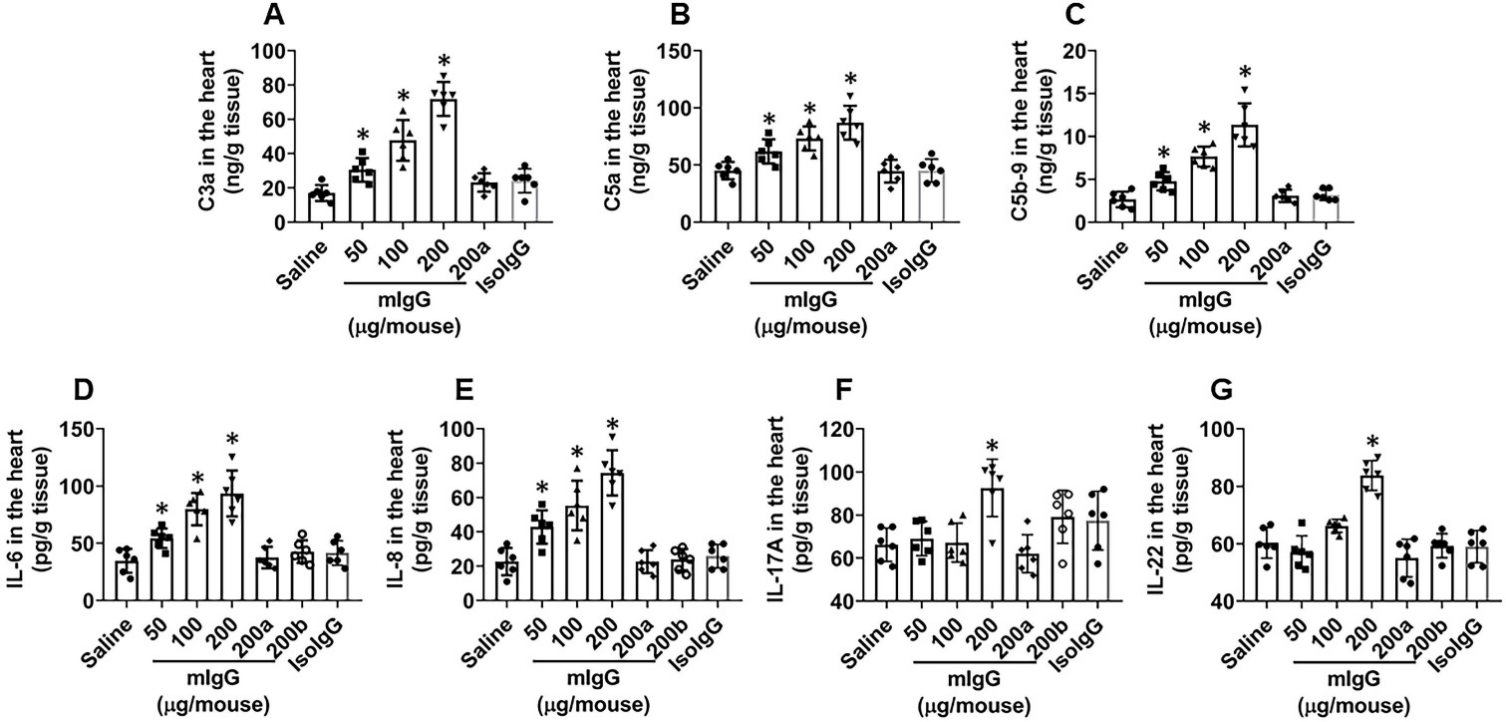
** mIgG induces complement activation and increases proinflammatory cytokine levels in the heart tissues**. Mice (6 mice per group) received mIgG or isotype IgG (control) through tail vein injection at indicated doses daily for 7 days. **A-C**, levels of C3a, C5a and C5b-9 in heart tissue extracts (by ELISA). **D-E**, levels of proinflammatory cytokines in heart tissue extracts (by ELISA). a, KRT10-deficient mice. b, CVF (200 U/kg) was peritoneally injected to mice 30 min before mIgG injection. Data of bars are presented as mean ± SEM. Each dot in bars presents data obtained from an individual sample. Statistics: ANOVA + the Tukey's multiple comparison test. *p<0.01, compared to the saline group.

The data reported above show that mIgG forms immune complexes with endothelial cells in the heart. Since immune complexes can activate complements 14, we next assessed the effects of mIgG on activating complements in the heart. Heart tissues were excised after treating with mIgG as described in Figure 4 and processed to assess the complement levels. The results showed that the levels of C3a, C5a and C5b-9 were detected in the hearts of mice treated with mIgG, which were abolished by the depletion of KRT10 expression (Figure 5A-C). In addition, we also detected the increase in levels of proinflammatory cytokines, including IL-6, IL-8, IL-17A and IL-22, in the heart tissues after exposure to mIgG, this was abolished by depleting the KRT10 expression or blocked by the presence of CVF, an inhibitor of complements 24 (Figure 5D-G); exposure to isotype IgG did not alter the levels of C3a, C5a, C5b-9, IL-6, IL-8, IL-17A and IL-22 (Figure 5A-G). The results thus demonstrate that exposure to mIgG can activate complement C3a, C5a and C5b-9, and increase inflammatory cytokines, including IL-6, IL-8, IL-17A and IL-22, in the heart tissues.

### mIgG induces neutrophilic inflammation in the heart

**Figure 6:**
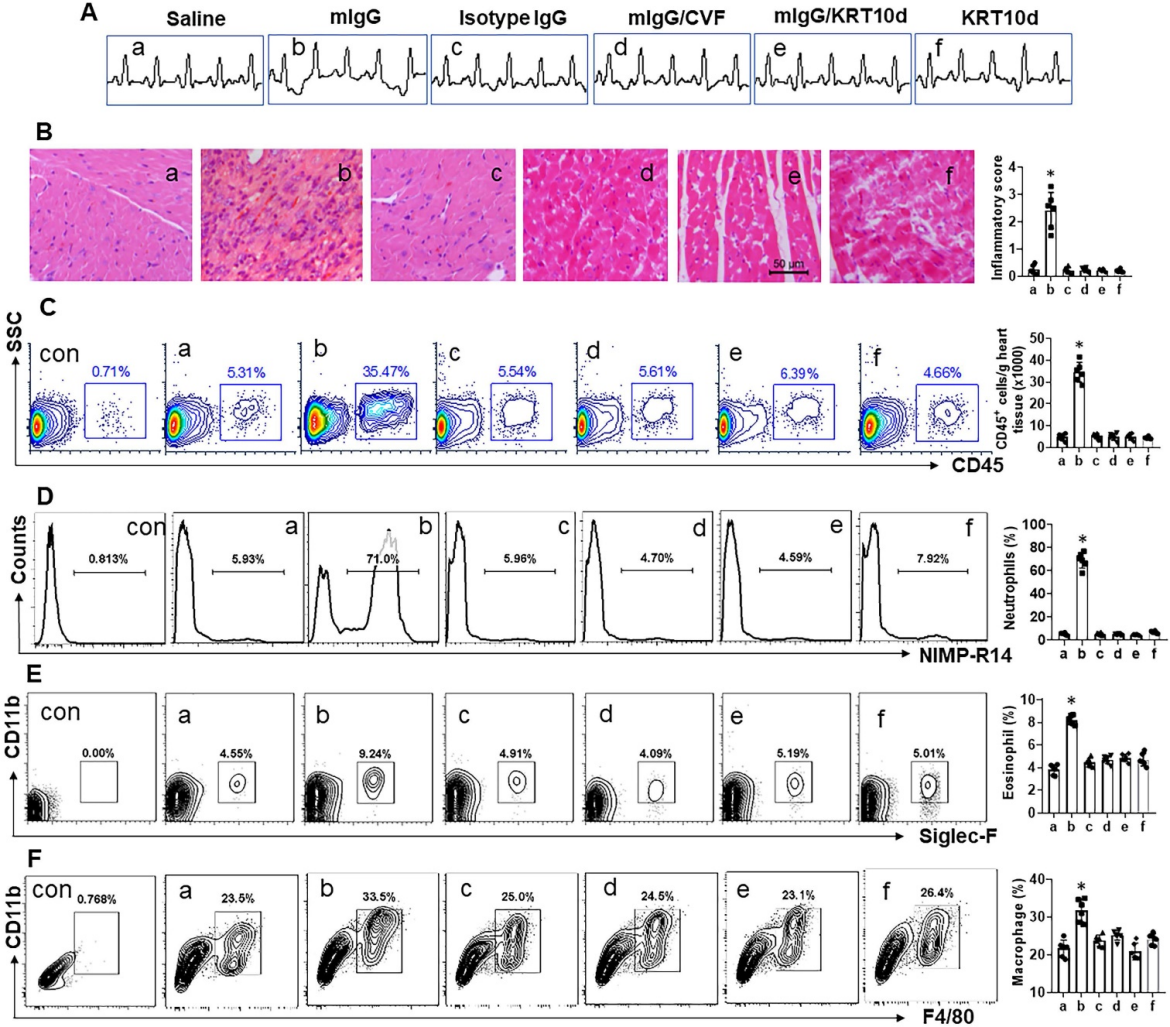
** mIgG induces neutrophilic inflammation in the heart**. Mice were treated with the procedures denoted above each subpanel of **A**. KRT10d: KRT10-depletion mice. A, representative ECG graphs. **B**, representative heart tissue histology images (original magnification: ×200). **C**, gated cells show the frequency of CD45^+^ white blood cells in the heart tissues. **D-F**, gated plots show the frequency of neutrophils (D), eosinophils (E) and macrophages (F) in the gated cells of panel C. Bar graphs show summarized data of subpanels on the left side. NIMP-R14: A mouse neutrophil marker. Siglec-F: A mouse eosinophil marker. Data of bars are presented as mean ± SEM. Each dot in bars presents data obtained from one mouse. *p<0.05, compared with the group a. Con: Isotype IgG staining.

**Figure 7:**
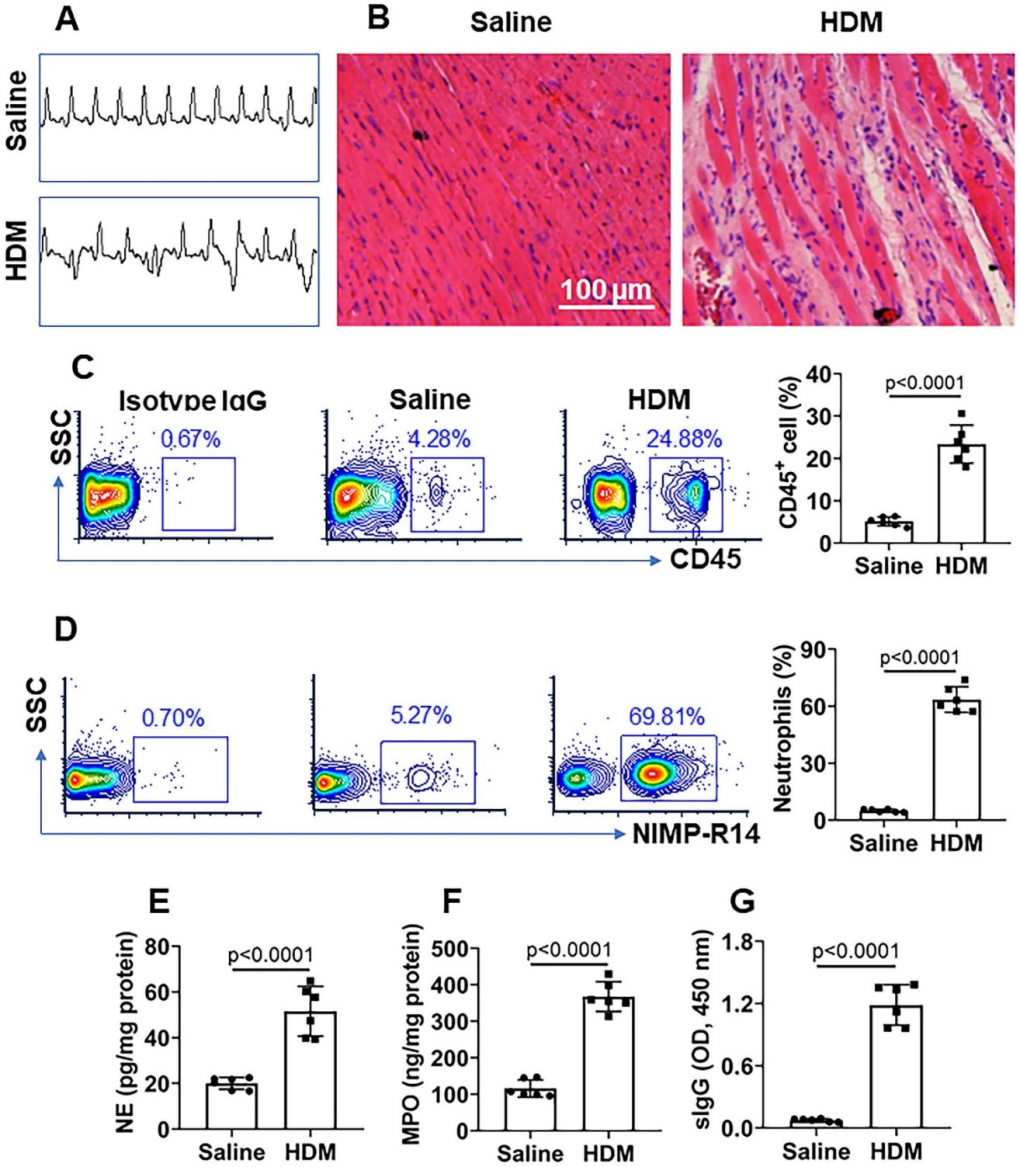
** HDM induces neutrophilic inflammation in the heart**. Mice were immunized with HDM and Freund adjuvant (control mice were treated with saline). **A**, representative ECG images show aberrant heart function in the mice. **B**, representative histology images show inflammatory condition in the heart. **C**, representative flow cytometry plots show CD45^+^ cell frequency in isolated mononuclear cells; the bars show summarized CD45^+^ cell frequency. **D**, representative flow cytometry plots show neutrophil frequency in CD45^+^ cells; the bars show summarized neutrophil frequency in CD45^+^ cells. **E-G**, neutrophil elastase (NE) levels (E), myeloperoxidase (MPO) levels (F) and HDM-specific IgG (sIgG) (G) in heart protein extracts. Data of bars are presented as mean ± SEM. Each dot presents data obtained from one sample. Statistics: *t* test. Each group consists of 6 mice.

Mice were treated with mIgG through tail vein injection daily for 1 week. After the sacrifice, the hearts were excised and processed for histology examination. We observed that treatment with mIgG resulted in heart dysfunctional ECG (Figure 6A), significantly increasing the infiltration of white blood cells in the heart tissues (Figure 6B-C). To differentiate the phenotypes of inflammatory cells in the heart tissues, mononuclear cells were isolated from the heart tissues and analyzed by flow cytometry. The results showed that neutrophils, eosinophils and macrophages were increased in the heart tissues after treatment with mIgG; among that, neutrophils were conspicuously increased (Figure 6D-F). The inflammatory status in the heart was abolished by depleting the KRT10 expression or administration of CVF, while the depletion of KRT10 alone did not induce heart inflammation (Figure 6A-F). In addition, the treatment with mIgG also induced inflammation in the lung and the colon (Figure S4). The results demonstrate that exposure to mIgG can induce neutrophilic inflammation in the heart. Moreover, by immunizing with HDM and Freund adjuvant, neutrophilic inflammation was also induced in the mouse's heart. We found aberrant ECG in immunized mice (Figure 7A); profound inflammatory cell infiltration in the heart tissues, in which neutrophils occupied 70% in CD45^+^ cells (Figure 7B-C). The contents of neutrophil elastase (NE), myeloperoxidase (MPO) and HDM-specific IgG (sIgG) in heart protein extracts were higher in immunized mice than those in control mice (Figure 7D-F).

## Discussion

It has been recognized that autoimmunity is associated with the pathogenesis of Mcd 7 while the underlying mechanism remains to be elucidated. This study revealed a factor, HDM specific autoantibody, could induce inflammation in the heart. The data show that HDM specific autoantibodies were detected in the serum of Mcd patients, in which the KRT10-specific IgG levels (mIgG) were conspicuously higher. The mIgG specifically bound the heart endothelial cells, activated complements and induced inflammation in the heart, which were verified by immunizing mice with HDM, in which the neutrophilic inflammation was also induced in the heart.

The pathogenesis of Mcd is to be further understood 5. Many studies suggest that viral infection plays roles in Mcd 3. In the recent years, the role of autoimmunity in the pathogenesis of a Mcd has been recognized 5. However, the causative factors in the initiation of autoimmune activities in Mcd are still unknown 5. The present data show that HDM-derived factors are associated with the pathogenesis of heart inflammation through inducing autoantibody production. As HDMs distribute extensively in human living environment, their secretions and body components can easily get into the human body. It is well known that HDMs are the major allergens involving the pathogenesis of allergic asthma, allergic rhinitis and allergic dermatitis 11. Higher titers of HDM-specific IgE are detected in the serum of patients with HDM allergy 25, indicating that HDM-specific immune response is induced in the body. As HDM-derived antigens can get into the body through inhalation, contact of the skin and ingestion by the digestive tract 20, it cannot rule out the probability that the ingested HDM-derived factors reach the heart and initiate an immune response. The reasoning is supported by the present data. We detected mIgG in the serum of Mcd patients. Others also noted such a phenomenon in patients with HDM-related allergies 13. Although some subclasses of IgG show protective effects in allergic diseases 13, the association between HDM-specific IgG and inflammation-induction has been recognized 15. Therefore, it is necessary to define the role of HDM-specific IgG in the pathogenesis of relevant diseases in human.

The data show high levels of KRT10-specific IgG in the serum of Mcd patients. KRT10 gene encodes KRT10 protein that is a constitutive protein in epithelial cells and some endothelial cells 26. Such an IgG responded to both human KRT10 and HDM KRT10 because of the high homologous gene sequences of KRT10 between HDM and human. In this regard, the KRT10-specific IgG originally against HDM KRT10 has the probability to attack human KRT10. Indeed, the present data show that the HDM KRT10-specific IgG recognizes the KRT10 in cardiovascular endothelial cells. Such recognition resulted in the formation of an immune complex of mIgG and the KRT10 in the endothelial cells. It is known that immune complexes can activate complement 17. Activated complements initiate inflammation in the local tissues by chemoattracting neutrophils 17. Such immune complexes were also found in the endothelial cells of Mcd heart tissues as shown by the present data, implying that HDM-derived factors may be involved in the pathogenesis of Mcd.

The endothelial barrier integrity is crucial in the maintenance of the homeostasis of cardiovascular system 27. Such as the endothelial barrier dysfunction is associated with the pathogenesis of hypertension 28, atherosclerosis 29 and pulmonary injury 30. Whether the endothelial barrier dysfunction is associated with heart diseases, such as Mcd, needs to be investigated. Factors impairing the endothelial barrier remain to be further elucidated. The present data demonstrate that HDM-derived factors are associated with the cardiovascular endothelial barrier dysfunction. There are plenty of proteins with competent antigenicity in the blood stream, which may penetrate the deep regions of the vessel tissues to initiate aberrant immune reactions. Such a phenomenon has been well recognized in other system; such as our previous studies which show that exposure to microbial products increases claudin-2 expression to compromise intestinal epithelial barrier function 31; and that chronic stress activates mast cells to induce colon epithelial barrier dysfunction 32. By employing FITC-labeled macromolecular tracers, we were able to test the endothelial barrier integrity of the heart *in vivo*. We found that exposure to mIgG markedly impaired the barrier functions in the heart. Since the barrier dysfunction may result in inflammation in the local tissues 33, mIgG may be a factor associating with inflammation in the heart.

One of the features of heart inflammation is the profound infiltration of inflammatory cells in the heart tissues. Previous studies showed such a phenomenon; high amounts of inflammatory cells were found in the heart tissues of Mcd patients as well as mice with experimental inflammation 9. In the present study, we also found abundant inflammatory cells in the heart tissues of Mcd patients. The inflammatory cells releasing bioactive substances can be responsible for the tissue injuries in the heart. The causative factors of heart inflammation include viral infection, rheumatic carditis, Trypanosoma cruzi, and bacterial infections 5. However, many questions are to be answered, such as, what are the triggering points to initiate the inflammation in the heart. The present data provide a plausible explanation for this point. We found that the mIgG bound to the endothelial cells in the heart. There was also evidence that complements were activated in the heart tissues. It is known that the activated complements are chemoattractant to inflammatory cells 14, which release inflammatory mediators, such as IL-6, IL-8, IL-17A and IL-22, to attract and activate neutrophils 34 to the local tissues and to induce inflammation.

The present data show that HDM-related IgG antibody is associated with the Mcd pathogenesis. It is known that some Mcd patients do not have appreciable Mcd symptoms until autopsy after death; for instance, about 20% sudden death people showed Mcd signs in the heart; autopsies revealed that about 0.12-12% patients who died of other diseases showed Mcd-like inflammation in the heart 1, 2. Therefore, it is of importance to have HDM-sensitized asthma patients examined, to see whether they also suffer from Mcd-like lesion, since about 0.65-1.3% people suffer from allergic asthma in the world, among which half of them are sensitized to HDM 11.

In summary, mIgG (the HDM KRT10-specific IgG) was found in the serum of Mcd patients. The mIgG bound to heart vascular endothelial cells to impair the barrier functions. By forming complexes with KRT10 in endothelial cells, mIgG activated complements, attracted inflammatory cells and induced inflammation in the heart. Therefore, HDM-derived factors may be an important factor associated with the pathogenesis of inflammation in the heart, such as Mcd.

## Supplementary Material

Supplementary materials and methods, figures.

## Figures and Tables

**Table 1:** Baseline characteristics of the myocarditis patients

Characteristics	myocarditis group	DCM group
Number	30	30
**Demography**		
Age (year)	29.2 ± 8.7	31.4 ± 6.8
Male (%)	21 (70%)	15 (50%)
Body-mass index	26.8 ± 9.5	28.2 ± 6.5
**Clinical characteristics**		
Diagnosis to transplantation (months)	18.0 ± 6.3	22.0 ± 11.4
SBP (mmHg)	102.3 ± 21.2	114.7 ± 15.9
Heart rate	92 ± 21	103 ± 19
**Medication history**		
Antiplatelet (%)	12 (40%)	9 (30%)
Digoxin (%)	24 (80%)	21 (70%)
ACEI/ARB (%)	15 (50%)	12 (40%)
Diuretic (%)	30 (100%)	30 (100%)
Beta blockers (%)	27 (90%)	24 (60%)
Antiarrhythmic (%)	21 (70%)	12 (40%)
**Cardiac function**		
Ⅰ (%)	0	0
Ⅱ (%)	3 (10%)	0
Ⅲ (%)	9 (30%)	3 (10%)
Ⅳ (%)	18 (60%)	27 (90%)
Hypertension	9 (30%)	6 (20%)
Diabetes	0	0
hyperlipidemia	6 (20%)	9 (30%)
**ECG**		
AF	12 (40%)	9 (30%)
LBBB	12 (40%)	3 (10%)
RBBB	9 (30%)	6 (20%)
Paroxysmal ventricular tachycardia	6 (20%)	12 (40%)
**UCG**		
left atrium diameter (mm)	51.2 ± 9.4	54.6 ± 12.5
LVEDD (mm)	62.8 ± 10.2	63.5 ± 14.3
EF (%)	37.6 ± 10.4	29.3 ± 9.8
Moderately to severe MR (%)	15 (50%)	21 (70%)

ECG: electrocardiogram; AF: atrial fibrillation; LBBB: left bundle branch block; RBBB: right bundle branch block; LVEDD: Left ventricular end diastolic diameter; EF: Ejection fraction; MR: Mitral regurgitation.
