# Supplementary material for: House Dust Mite Specific Antibodies induce Neutrophilic Inflammation in the Heart
Source: Theranostics. 2020 Jul 9;10(19):8807–17. doi: 10.7150/thno.47134 (PMC7392007; doi:10.7150/thno.47134)
Supplement: Supplementary file 1 — Supplementary materials and methods, figures. [file thnov10p8807s1.pdf]

Supplemental materials

Reagents

KRT10 RNAi kit, antibodies of CD45 (Alexa Fluor® 488), NIMP-R14 (Alexa Fluor® 546), Siglec-F (Alexa Fluor® 594), F4/80 (Alexa Fluor® 647), MPO and KRT10 were purchased from Santa Cruz Biotech (Santa Cruz, CA). ELISA kits of IgG, IgE, MPO, C3a, C5a, C5b-9, IL-6 and IL-8 were purchased from R&D Systems (Minneapolis, MN). FITC-labeling kit, FITC-dextran and ChIP kit were purchased from Sigma Aldrich (St. Louis., MO). Reagents and materials for RT-qPCR, Western blotting and immunoprecipitation were purchased from Invitrogen (Carlsbad, CA).

Preparation of mononuclear cells with heart tissues

Human hearts and mouse hearts were collected and cut into small pieces. The samples were incubated with collagenase IV at 1 mg/ml for 2 h at 37 °C with mild agitation. Single cells were collected by passing the samples through a cell strainer (70 µm first, then 40 µm). The cells were then pelleted by Percoll gradient density centrifugation. Viability of the isolated cells was greater than 98% as assessed by Trypan exclusion assay.

Immune cell isolation

Immune cells were labeled with relevant reagent kits and isolated by flow cytometry cell sorting (FCCS). Purity of isolated cells was greater than 95% as assessed by flow cytometry.

Assessment of mite-specific antibodies in the serum

Blood samples were collected from Mcd patients and healthy control (HC) subjects. The sera were isolated. Mite-specific antibodies (IgG and IgE) in the sera were evaluated by enzyme-linked immunosorbent assay (ELISA). Briefly, microplates were coated with mite proteins and stayed at 4 °C overnight. After washing with Tris-buffered saline containing 0.1% Tween 20 (TBST) for 3 times, serum samples were added to the plates in triplicate. The plates were kept at 4 °C overnight, washed with TBST for 3 times, incubated with peroxidase-labeled secondary antibodies for 2 h at room temperature. The plates were developed with TMB and then read with a microplate reader.

Isolation of HDM-specific IgG from the sera of Mcd patients

The sera collected from Mcd patients were treated with protein G agarose beads to precipitate IgG. HDM-specific IgG was purified from the IgG by affinity chromatography (the column was filled with sepharose beads absorbed with HDM antigens).

Purification of KRT10 protein from the heart tissues

Proteins were extracted from the explant hearts. KRT10 protein was purified from the heart proteins by affinity chromatography (the column was filled with sepharose beads absorbed with anti-KRT10 antibody).

To define the heart proteins recognized by HDM-specific IgG

Proteins extracted from heart tissues were mixed with purified HDM-specific IgG at a ratio of 1:1 (w/w) at room temperature for 30 min to form immune complexes. The complexes were then precipitated by incubation with protein G agarose beads for 1 h. The beads were collected by centrifugation at 3,000 *g* for 10 min. Proteins on the beads were eluted with an eluting buffer and dissociated from the IgG. After removing the IgG by protein G, remained proteins were analyzed by mass chromatography.

Assessment of mIgG binding endothelial cells of the heart

HUVEC cells or heart tissue sections were fixed with 1% paraformaldehyde for 30 min and stained with HDM-specific anti-KRT10 antibodies. On the other hand, the HDM-specific anti-KRT10 antibodies were added to the culture of HUVEC cells, or treated mice through tail vein injection daily for 6 days. The cells were collected 30 min later, fixed with 1% paraformaldehyde for 30 min. Heart tissue sections were prepared and fixed with acetone. The HUVEC cells and heart tissue sections treated with both procedures were stained with fluorescence-labeled secondary antibodies for 1 h at room temperature, mounted with cover slips, and observed with a confocal microscope (LSM710).

Assessment of the effects of mIgG on heart endothelial barrier integrity

HUVEC monolayers were prepared in a transwell system. Upon confluence of the monolayer, mIgG was added to the culture at 0, 2.5, 5 and 10 µg/ml, respectively, in the presence of complement-containing serum (1% of culture medium). Transepithelial electrical resistance (TEER) of the monolayer was recorded at 0 and

24 h, respectively. The resistance across the epithelial monolayer was recorded using a MILLICELL-ERS (Millipore Corporation, Bedford, MA, USA) with a pair of "chopstick" electrodes, according to our established procedures (Huang H, Liu JQ, Yu Y, Mo LH, Ge RT, Zhang HP, Liu ZG, Zheng PY, Yang PC. Regulation of TWIK-related potassium channel-1 (Trek1) restitutes intestinal epithelial barrier function. Cell Mol Immunol. 2016; 13: 110-8). FTIC-labeled dextran was added to the upper chambers (1 mg/ml) at 24 h time point. One hour later, samples were taken from basal chambers of transwell. On the other hand, after treating mice with mIgG daily for 6 days, FITC-dextran was injected into the mice through tail vein at 10 mg/kg. Mice were sacrificed 1 h later. The heart was lavaged with saline through venae cava inferior to wash out the residue FITC-dextran within the heart blood vessels. The heart tissues were cut into small pieces, immersed in a protein extracting buffer and homogenized with a homogenizer at 4 °C. The lysates were centrifuged at 10,000 *g* for 10 min. Supernatant was collected. Levels of FITC in the supernatant and the samples collected from basal chambers of transwell were determined by fluorescent spectrometry and regarded as an indicator of the functional status of endothelial barrier.

Flow cytometry

Cells were collected from relevant experiments. In the surface staining, cells were stained with fluorescence-labeled antibodies of interest or isotype IgG at 4 °C for 30 min. In the case of intracellular staining, cells were treated with fixative containing 0.1% triton X-100 for 1 h, followed by incubation with fluorescence-labeled antibodies of interest or isotype IgG for 30 min at 4 °C. After washing with PBS

thoroughly, cells were analyzed with a flow cytometer. The data were analyzed with flowjo. Data from isotype IgG staining were used as a gating reference.

Preparation of protein extracts

Tissues from explant hearts were cut into small pieces and homogenized in a homogenizer in an ice bath. Cells were collected from relevant experiments. The cells and tissues were lysed with the RIPA lysis buffer. Lysates were centrifuged at 10,000 *g* for 10 min. Supernatant was collected and used as the cytosolic extracts. The pellets were resuspended in a nuclear lysis buffer and stayed for 30 min. The lysates were centrifuged at 10,000 *g* for 10 min. Supernatant was collected and used as the nuclear extracts. All the procedures were performed at 4 °C.

Western blotting

Proteins were fractioned by SDS-PAGE and transferred onto a PVDF membrane. After blocking by 5% skim milk, the membrane was incubated with the primary antibodies of interest overnight at 4 °C, washed with TBST (Tris-buffered saline containing 0.1% Tween 20) 3 times, incubated with peroxidase-labeled secondary antibodies for 2 h at room temperature, washed with TBST 3 times. Immunoblots on the membrane were developed with ECL and photographed in an imaging device.

Generation of mite KRT10-specific polyclonal antibodies (IgG)

The customized Mite KRT10 proteins were provided by Shanghai Sangon Biotech (Shanghai, China). To generate KRT10-specific antibodies, BALB/c mice were immunized with KRT10 protein (0.1 mg/mouse) mixed with 0.1 ml completed Freud

adjuvant on day 0, day 3 and day 7, respectively. The mice were boosted by injection with KRT10 protein (0.1 mg/mouse) every other week 4 times with incomplete Freud adjuvant. After the immunization complete, blood was collected from the mice through pulling out the eyeballs, the sera were isolated. KRT10-specific antibodies were purified from the sera by affinity chromatography and characterized by ELISA (Fig. S1).

Mice

Male C57BL/6 mice (6-8-week-old) were purchased from the Beijing Experimental Animal Center (Beijing, China). The KRT10 knockout mice were provided by the Animal Institute of Chinese Agricultural Academy (Beijing, China) (Fig. S2 in supplemental materials). Mice were maintained in a specific pathogen free facility with accessing food and water freely. The animal experimental procedures were approved by the Animal Ethical Committee at Beijing Fuwai Hospital.

Cell culture

HUVECs were purchased from ATCC (Manassas, VA) and cultured in dulbecco's modified eagle medium. The medium was supplemented with 10% fetal calf serum, 100 U/ml penicillin, 0.1 mg/ml streptomycin and 2 mM L-glutamine. The medium was changed daily. Cell viability was greater than 99% as assessed by Trypan blue exclusion assay and flow cytometry with both Annexin V and propidium iodide staining (Fig. S5).

Mouse electrocardiography (ECG)

Mice were anesthetized by intraperitoneally injecting with diazepam (10 mg/kg). The DII derivation-transducers were set subcutaneously. ECG was recorded with an ECG device (The digital Power Lab 2/20 Systems; PanLab Instruments, Spain). The data were analyzed with the Scope software package (PanLab instruments).

Inflammatory scores

Mouse heart tissue inflammation was evaluated with a 0-4 scoring system: 0 = no noticeable inflammatory signs; 1 = 1-5 foci with mononuclear cell infiltration in a microscopic field (×200); 2 = more than 5 foci with mononuclear cell infiltration in a field; 3 = profound mononuclear inflammation involving over 20 % of the area, without necrosis; and 4 = profound inflammation with necrosis. The scoring process was carried out blindly to avoid the observer bias.

Immunohistochemistry

Frozen heart tissue sections were prepared and fixed with cold acetone for 20 min. The sections were blocked by 1% bovine serum albumin (BSA) for 30 min, incubated with primary antibodies of interest or isotype IgG overnight at 4 °C, washed with phosphate buffered saline (PBS) 3 times, incubated with fluorescence-labeled secondary antibodies for 2 h at room temperature and washed with PBS 3 times. The sections were contrast stained with propidium iodide to show the nucleus. After mounting with cover slips, the sections were observed under a confocal microscope.

Labeling IgG with FITC

The generated IgG was labeled with FITC with a reagent kit (Sigma Aldrich) following the manufacturer’s instruction.

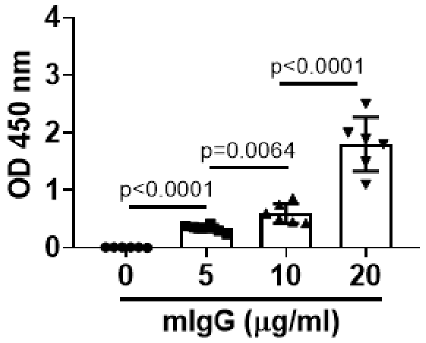

Figure S1. Generation and identification of mIgG. Polyclonal anti-mite antibodies were generated by immunizing New Zealand rabbits with HDM and complete Freud adjuvants. The mIgG was verified by ELISA by the “HDM-mIgG-anti-IgG Ab” strategy. The bars show mIgG dose-response levels. Data are presented as mean ± SEM. Each dot in bars presents data obtained from one sample. Statistics: ANOVA + Bonferroni test. The data represent 6 independent experiments.

Generation of endothelial KRT10-knockout (KO) mice

The KRT10 gene was specifically knocked out in endothelial cells of mice through genetic engineering approach. To avoid affect the role of KRT10 in other cells, KRT10 was specifically deleted from endothelial cells with CD34 as the specific marker. Following published procedures (J Clin Invest 1996; 98: 600-3; Elife 2014; 3: e01949), we generated mice with loxP-flanked KRT10 gene (flox). In the first step, we constructed a gene targeting vector containing three loxP sites, in which two of them

flanking the neomycin resistance gene. The genomic locus was modified between vector and KRT10 gene in embryo stem (ES) cells by homologous recombination. The loxP-flanked KRT10 gene was targeted by CD34-Cre expression in ES cells. Using the modified ES cells, a loxP-KRT10 containing mouse line was generated. Then, we crossed the mouse strain harboring two loxP sites in the KRT10 gene with another strain expressing CD34-Cre recombinase. Thus, only in cells expressing CD34-Cre the KRT10 gene becomes inactivated but remained active in other cells of the body. Besides expressing in endothelial cells, CD34 is also expressed in other cells, such as hematopoietic stem cells, the Cre gene was modified to be functional only in the ribosomes, but could not penetrate the nuclear membrane. Thus, the loxp-KRT10 gene was remained intact in the mice. One week before the experiments, the mice were treated with tamoxifen citrate salt (Sigma Aldrich, St. Louis, MO; 0.3 g/kg in 0.2 ml 10% Etoh in corn oil) by gavage-feeding daily for five days. Control mice were received oral gavage of 0.2 ml 10% EtoH in oil. Tamoxifen increases the permeability of the nuclear membrane to allow the CD34-Cre penetrate the nuclei to cleave the flox-KRT10 gene. As assessed by flow cytometry, all CD34^{+} endothelial cells in the heart did not express the KRT10 (Fig. S2).

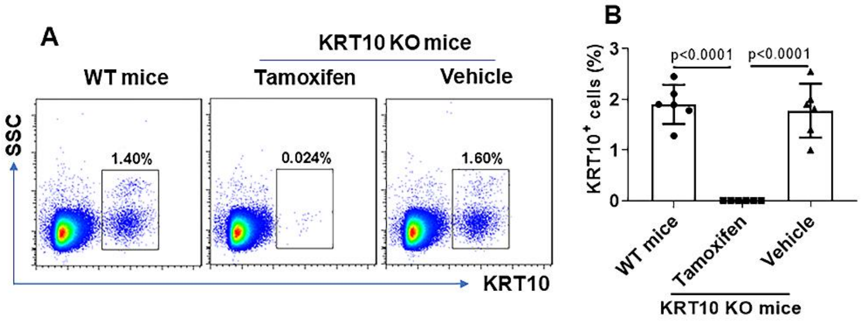

Figure S2. Depletion of KRT10 in endothelial cells in mice. Mice with KRT10 gene KO endothelial cells were developed as described above. The heart was excised from each mouse upon the sacrifice. Single cells were prepared with the heart tissues, stained with anti-KRT10 antibody and analyzed by flow cytometry. A, the gated dot plots show the frequency of KRT10^{+} cells. B, bars show summarized KRT10^{+} cells in the heart. Tamoxifen (or vehicle): KO mice were treated with tamoxifen (or vehicle) as described above. Each group consists of 6 mice. The data of bars are presented as mean ± SEM. Each dot in bars presents data obtained from one mouse. Statistics: ANOVA + Bonferroni test.

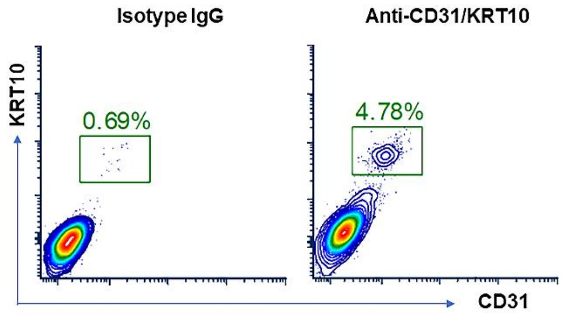

Figure S3. CD31^{+} endothelial cells in the heart express KRT10. Heart samples were excised from naïve mice. Single cells were prepared from the heart tissues and analyzed by flow cytometry. The gated dot plots show CD31^{+} KRT10^{+} cells. The data indicate that heart endothelial cells also express KRT10. The data represent 6 independent experiments.

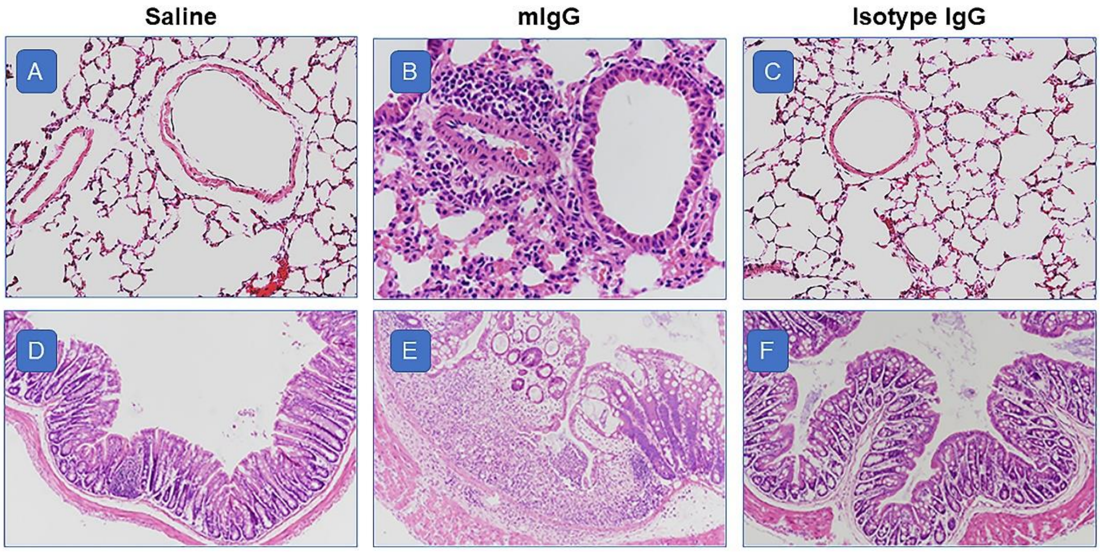

Figure S4. mIgG induces inflammation in the lung and intestine. Related to Fig. 6 in the main text. C57BL/6 mice were treated with saline or mIgG (20 µg/mouse in 0.1 ml saline) or isotype IgG through tail vein injection daily for 6 days. Mice were sacrificed on day 7. A piece of the lung tissues and a piece of intestinal tissues were processed to histology examination. A-C, representative lung tissue histology images. D-E, representative colon tissue histology images. Each group consists of 6 mice.

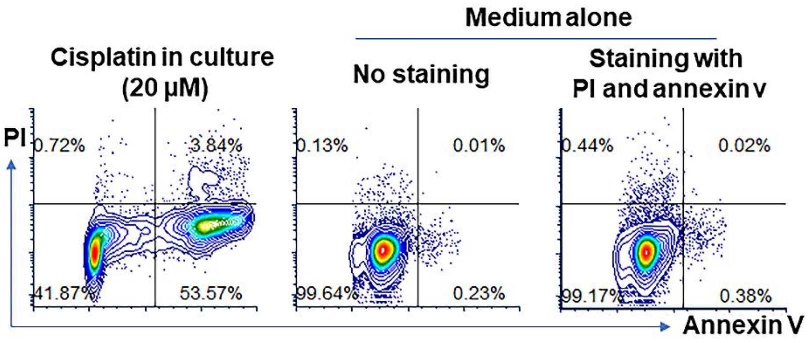

Figure S5. Cell viability assessment. Cell viability was assessed by flow cytometry with annexin v and PI (propidium iodide) staining. Cisplatin (an apoptosis inducer; Sigma Aldrich) was added to the culture of a portion of cells overnight used as positive control. Representative flow cytometry plots show staining results. The data represent 3 independent experiments.
